# Acetylsalicylic Acid–Primus Inter Pares in Pharmacology

**DOI:** 10.3390/molecules27238412

**Published:** 2022-12-01

**Authors:** Łukasz Fijałkowski, Magdalena Skubiszewska, Grzegorz Grześk, Frankline Kiptoo Koech, Alicja Nowaczyk

**Affiliations:** 1 Department of Pharmacometrics and Molecular Modeling, Faculty of Pharmacy, Collegium Medicum in Bydgoszcz, Nicolaus Copernicus University, 2 dr. A. Jurasza St., 85-094 Bydgoszcz, Poland; 2 Department of Cardiology and Clinical Pharmacology, Faculty of Health Sciences, Ludwik Rydygier Collegium Medicum in Bydgoszcz, Nicolaus Copernicus University, 75 Ujejskiego St., 85-168 Bydgoszcz, Poland; 3 School of Pharmacy, Kabarak University, Nairobi P.O. Box 20157, Kenya

**Keywords:** aspirin (ASA), ASA formulations, ASA derivatives

## Abstract

Acetylsalicylic acid (ASA) is one of the first drugs to be obtained by synthesis while being the most used. It has experienced the longest lasting commercial success and is considered the most popular drug of the modern era. ASA, originally used as an anti-inflammatory medication, nowadays is predominantly used as an antiplatelet agent for prophylaxis in cardiac patients. Many studies show that the benefits of using ASA far outweigh the potential risk of side effects. With particular emphasis on the possibility of ASA repositioning for new therapies, extending the indications for use beyond the diseases from the spectrum of atherosclerotic diseases, such as cancer, requires shifting the benefit–risk ratio, although very good, even more towards safety. Interesting activities consisting not only of changing the formulation but also modifying the drug molecule seem to be an important goal of the 21st century. ASA has become a milestone in two important fields: pharmacy and medicine. For a pharmacist, ASA is a long-used drug for which individual indications are practically maintained. For a doctor, acetylsalicylic acid is primarily an antiplatelet drug that saves millions of lives of patients with coronary heart disease or after a stroke. These facts do not exempt us from improving therapeutic methods based on ASA, the main goal of which is to reduce the risk of side effects, as well as to extend effectiveness. Modified acetylsalicylic acid molecules already seem to be a promising therapeutic option.

## 1. Introduction

Aspirin (ASA, acetylsalicylic acid ATC code: N02BA01 [1], DrugBank ID: DB00945 [2], brand names: Arthritis Pain, Aspi-Cor, Aspirin 81, Aspirin-Low, Bayer Plus, Bufferin, Ecortin, Eciprin, Miniprin, Vazalore [3]), is one of the first drugs to be obtained by synthesis. It is regarded as, being the most used drug with the longest lasting commercial success [4]. ASA was originally used predominantly as an anti-inflammatory medication [5]. Nowadays, ASA is still a favorite of patients with a consumption of 44,000 tons of ASA each year, which is equivalent to approximately 120 billion aspirin tablets [6].

So far, there have been a significant number of publications written about ASA—we have focused on the uniqueness of the structure—and want to bring it closer to the reader. The use of aspirin-like drugs in modern medicine is very broad and includes the treatment of inflammation, pain and various cardiovascular diseases [7]. Many studies show that the benefits of using ASA far outweigh the potential risk of side effects. Like other medicines, ASA is toxic in high doses (over 150 milligrams per kilogram of body weight) [8]. In addition, we outline the current research trends of aspirin derivatives with particular emphasis on the possibility of ASA repositioning for new therapies. Last, but not least, ASA is one of the few drugs that has been developed in each of the possible pharmaceutical formulations and according to our best knowledge this article is the first attempt to provide a concise description of all the formulations in various indications.

## 2. History of ASA

The first use of willow bark containing salicin as analgesic was described in the Ebers Papyrus in 1534 BC [9]. Hippocrates also used an infusion of willow bark to treat pain. The key precursor for the synthesis of ASA was salicylic acid (SA). SA was chemically described and synthesized in 1859 by Hermann Kolbe [10,11]. However, the pharmacological use of SA was significantly limited due to its side effects such as nausea, gastric irritation and tinnitus. An important step in history of ASA was first synthesis of pure and stable form achieved by Felix Hoffmann [12]. The date of this synthesis (10 August 1897) is considered as the birthday of ASA [13]. Sales of ASA in tablet form began in 1904 and contributed to immediate commercial success of these drugs. It is worth emphasizing that ASA is one of the first industrial drugs available in the form of tablets in the world. On 1 February 1899, Bayer registered the trademark name in Berlin. ASA was then patented (patent no. 644077) in the United States in 1900. After this, ASA started its great triumph and became the most popular painkiller worldwide [14]. Already in 1904, the annual production of ASA was 25,823 kg. The outbreak of World War I in 1914 interrupted the international trade in pharmaceuticals and caused the ASA to become available without prescription [15]. From this time, the history of ASA has been very rich. It seems that the breakthrough for this drug was the discovery of its anticoagulant activity. Lawrence Craven used ASA in primary cardiovascular prevention in 1953. ASA’s mechanism of action was discovered in 1971 by Vane, Samuelsson and Bergström who then received the Nobel Prize for the work [14,16].

A cursory search of the PubMed database reveals that 70,694 articles about ASA have been published, while Google Scholar indicates 1,360,000 scientific and medical papers on ASA. Nonetheless, ASA is still a very attractive drug for scientists who are looking for an antidote to new threats [17]. Even today, it is one of the most studied drugs in the world [18]. The U.S. Food and Drug Administration (FDA) Clinical Trials Register (CTR) databased found 2287 studies [19], while the EU CTR currently contains 431 clinical trials for ASA with the EudraCT protocol, of which 69 are clinical trials conducted in people under 18 years of age [20], and the Australian and New Zealand CTR databases found only 87 studies [21]. As mentioned above, another important factor is that this substance is produced in great amount, i.e., in 2020 year it synthesized approx. 44,000 tons of ASA [6]. In the United States alone, more than 50 million people regularly take 10 to 20 billion ASA tablets to help prevent cardiovascular disease (CAVDs) and celebrovascular disease (CEVDs) [6,22].

## 3. ASA Structure

**Figure 1:**
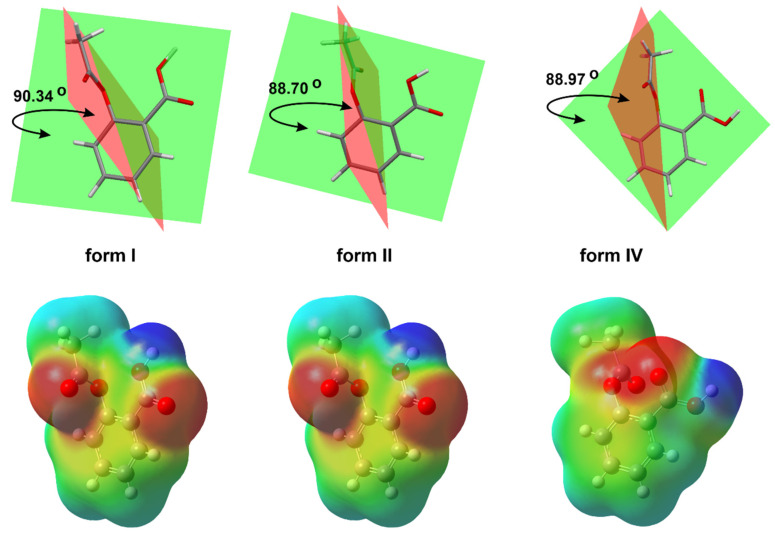
The basic crystal structure of ASA (CSD refcode ACSALA); compared 3D molecular view of the forms, I (CSD refcode ACSALA), II (CSD refcode ACSALA13) and IV (CSD refcode ACSALA24); the orientation of OH moiety with respect to the Ac group is on the near side in form I and II, but in form IV, we can observe the inverse arrangement of mentioned moieties; calculated molecular electrostatic potential map of ASA forms.

A total of 30 crystal structures of ASA are deposited in the Cambridge Structural Database (CSD) [23]. They have been assigned as ref codes from ACSALA to ACSALA29. The first crystal structure was obtained in 1964 [24] and was subsequently confirmed with greater accuracy about 20 years later [25] (Figure 1). ASA is O-acetyl (Ac) derivative of SA [26]. ASA has three bonds about which rotation is possible [27]. It was proven in computational study that form I has the lowest energetic minima [26]; due to this fact, it is the most stable ASA structure [28].

**Figure 2:**
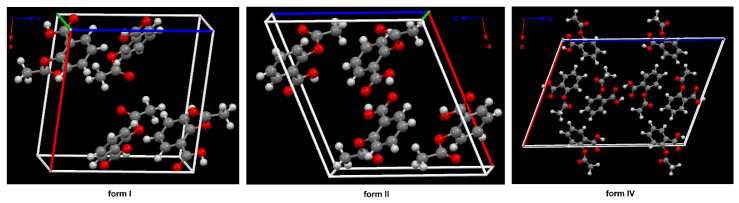
The crystal packing of ASA, form I (CSD refcode: ACSALA), form II (CSD refcode: ACSALA13) and form IV (CSD refcode: ACSALA24) along the b axis; formed through O–H ···O hydrogen bonds. The key difference between the structures I and II lies in the way the layers are arranged and bonded. Form I shows molecules in direct contact across the layer boundary forming the hydrogen bonded centrosymmetric acetyl groups (Ac) dimer. Form II shows the contraccatemericCO_2_H dimer and interlayer acid dimers are connected via catemeric methyl C–H ···O and phenyl C–H ···O hydrogen bonds interactions. The two arrangements are related to each other by a relative shift of adjacent layers along the crystallographic c axis in space group P21/c. The known crystal structure of ASA. In the structure of form IV, the plane of the Ac group is nearly perpendicular to the aryl ring plane, as in form I and II (see Figure 1). Color code: H = white, C = grey, O = red.

The phenomenon referred to as polymorphism relates to an organic molecule that can crystallize in more than one way or form [27,28,29,30]. Nowadays, it is assumed that ASA has four polymorphs [31]. From the pharmaceutical industry perspective, polymorph control is a very important issue due to the fact that each crystal form has different physical and chemical features, such as thermodynamic, kinetic, packing, surface, mechanical and spectroscopic properties. It determines the solubility, stability and bioavailability of the selected chemical as a drug [32]. In this way, the polymorphism directly affects the performance and functionality of the active ingredient in the drug product. The first crystal structure of form II was obtained in 2005 [33] (Figure 2).

The form III was crystallized from form I through the transformation at high pressures (2 GPa) and was identified by means of Raman spectroscopy in 2015 [34]. Nevertheless, ASA crystal form III has not been obtained so far. Additionally, upon release of the pressure, form III transforms back to form I, indicating that the high-pressure phase is not stable at ambient conditions. Form IV was crystallized from the melt, and its structure was determined using a combination of X-ray powder diffraction analysis and crystal structure prediction algorithms in 2017 [35]. Optimized structure of ASA form IV (CSD refcode: ACSALA24) indicates that the orientation of the OH group of the CO_2_H moiety is on the far side with respect to the Ac group (Figure 1) [35]. The computational studies have suggested n → π* interaction between the aromatic COOH and the -C=O carbon of the Ac group [36]. Even after the discovery of the crystalline structure of ASA form II, the existence of the polymorph is still controversial due to the unique mutual growth phenomenon observed between the two polymorphic domains. However, it is clear that the two polymorphs exhibit significantly different solid state properties such as dissolution rate, mechanical properties, crystal habit, melting point and pKa [37].

### 3.1. The Basic Physical, Chemical and Biological Properties of ASA

Transport of pharmaceuticals in biologic tissues includes solvation in and distribution between environments of different properties in terms of lipophilicity, basicity, etc. [38,39,40]. The drug solubility testing in solvents present in the body is necessary to determine bioavailability, and the dose of the drug that should be administered to produce a specific therapeutic effect. The solubility of drugs depends on the pH of the environment in which the drug is absorbed and on the dissociation coefficient-pKa [41]. The solubility of a biologically active compound in water, i.e., in the main component of every organism, is checked for almost every compound. Other important solvents are ethanol (a model responsible for the transport of the drug in the body) and octanol (a model lipid, a component of biological cell membranes). SA is among the drugs that have been best studied in terms of their solubility in both organic and inorganic solvents [42]. The solubility of this drug was tested in such solvents as water, methanol or acetic acid. SA is poorly soluble in water. However, its sodium salt dissolves very well. ASA is more soluble in ethanol, ethyl ether, chloroform, sodium hydroxide solution and sodium carbonate solution than in water. However, with the access of moisture, it undergoes hydrolysis to SA and AcOH. It easily decomposes in aqueous solutions, and an increase in pH significantly accelerates both dissolution of the compound in water and its decomposition [39]. Table 1 presents the basic chemical, physical and biological properties of ASA compared to selected non-steroidal anti-inflammatory drugs (NSAIDs).

The bioavailability of most medicinal substances in the lumen of the gastrointestinal tract (GI) is increased when the drug is in a non-ionized state. Only the undissociated molecule is lipophilic enough to pass through the stomach or intestinal wall by passive diffusion. Due to its chemical structure acetylsalicylic acid shows a pH-dependent equilibrium of the ionized (hydrophilic) and the non-ionized (lipophilic) form of the salicylate [50]. ASA is a weak acid that is poorly dissociated in setting the pH of gastric fluid, and therefore rapidly resorbed by various gastric cell membranes [51]. ASA dissociates to a greater extent in the intestine due to the higher pH in this part of the GI, thus improving its solubility [52]. The higher ionized rate determines the better absorption of ASA in the small intestine than in the stomach at the same pH range. At pH 3.5 or 6.5, the intestinal absorption of ASA is greater than the absorption of the compound in the stomach. The stomach does not absorb ASA at a pH of 6.5 (e.g., the gastric pH of infants ranges from 6 to 8).

### 3.2. Mechanism of Action for ASA

**Figure 3:**
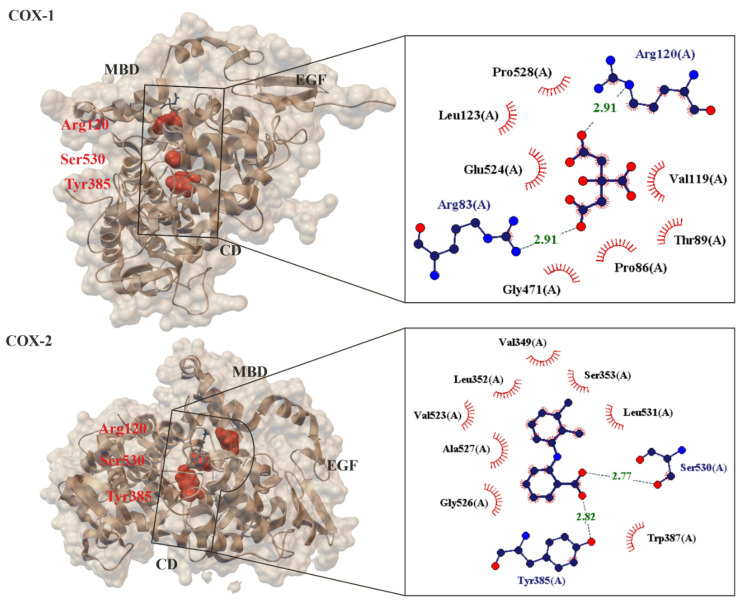
Comparison of 3-D crystal structures of human cyclooxygenase COX-1 (PDB 6y3c, 3.36 Å) [60] and COX-2 (PDB: 5ikr; 2.34 Å) [61] highlighting the bound ligands, important protein domains (such as EGF: epidermal growth factor domain, MBD: membrane binding domain and CD: catalytic domain) and amino acids (Arg120, Ser530, Tyr385). Enlarged area showing structural elements around acidic ligand binding site. Residues forming hydrogen bonds (dashed lines) with the crystalized ligand are shown in spherical form with interatomic distances in Å. Residues forming Van der Waals interactions with ligands are shown as labeled arcs with radial spokes pointing toward the ligand atoms.

At present, no one doubts that the known mechanisms of action for ASA fall into two categories, they are cyclooxygenase (COX, alternate name Prostaglandin H_2_ synthase PGHS, EC.1.14.99.1 [53]), i.e., COX-dependent and COX-independent pathways [54]. When ASA is absorbed into the blood, it breaks down into two pharmacologically important molecules: active acetate and salicylate [17]. From a chemical point of view, the primary mechanism of action for ASA is the transfer of the Ac group to -OH and -NH_2_ functionalities present in biological macromolecules [12]. It has been proven that ASA reacts with nucleophilic groups on proteins (such as Lys and Arg (with -NH_2_ group), Ser, Thr, Tyr (with -OH group, and Cys (with -SH group)) resulting in irreversible acetylation. From a biological point of view, the key mechanism of action for ASA is the irreversible inhibition of prostaglandin peroxide synthase 1 (COX-1), 2 (COX-2) and 3 (COX-3)) of the prostaglandin precursor and thromboxane producing enzymes [55,56,57]. COX is a membrane enzyme endowed with a long and narrow hydrophobic enzyme tunnel that runs from the surface of the membrane into the interior of the protein molecule [58] (Figure 3). COX-1 is structurally and enzymatically similar to COX-2 [59].

**Figure 4:**
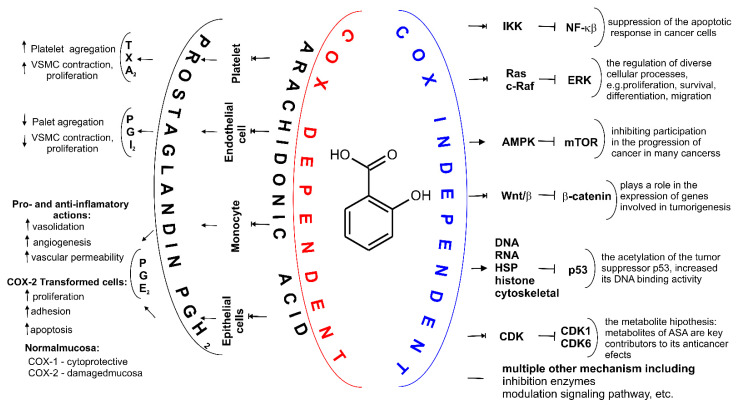
Two major mechanisms of action for ASA. Depending on the cell type COX-dependent mechanism affects synthesis of different prostaglandins (PGs) including PGH_2_, PGE2, PGF2α, prostacyclin PGI2 and tromboxane A2 (TXA2). Through various COX-independent mechanisms, ASA may modulate carcinogenesis and alter growth rate of the intestinal tumor. These pathways, including inhibition of nuclear factor (NF)-κB signaling and the extracellular signal-regulated kinase (ERK) signaling, cells and may arrest cell growth by inhibiting cyclin dependent kinase CDKs, inhibition of the Wing-like glycoproteins, i.e., Wnt signaling, β-Catenin phosphorylation, activation adenosine monophosphate activated protein kinase (AMPK), decreasing mechanistic target of rapamycin (mTOR) signaling, acetylation many proteins such as histones, cytoskeletal and heat shock proteins (HSP), glycolytic and pentose pathway enzymes, proteasomal subunits and mitochondrial proteins, proteins involved in translation, wild-type and mutant a tumor suppressor protein (p53), and multiple other mechanisms, e.g., inhibition hydroperoxy fatty acid peroxidase in the lipoxygenase pathway of arachidonic acid metabolism.

The small difference in size between the active sites of COX-1 and COX-2 has been exploited by pharmaceutical companies to develop selective COX-2 inhibitors, such as celecoxib [62], rofecoxib [63] and meloxicam [64,65], which reduce inflammation without damaging the stomach mucosa [66]. COX-3 is a splicing variant of COX-1 which has retained intron-1 during translation and which is found in human tissues in a polyadenylated form. Selective inhibition of COX-3 will discover potent and valuable new drugs for controlling pain and fever [66]. ASA acetylates SER-530 COX-1 thereby block prostaglandins (PGs) and thromboxane A2 (TxA2) synthesis in platelets and reduce platelet aggregation. A vasoconstrictor, TxA2, also aids in the aggregation of platelets during hemostasis (Figure 4). This mechanism of action explains the effect of ASA in preventing thrombosis of the coronary arteries and cerebral vessels [67,68]. These result in a decreased chance of thrombosis or thrombotic events, but the exact role of ASA in primary prevention in still uncertain [56,69].

ASA is the only NSAID that covalently modifies the COX enzymes. ASA binds to SER- 516 residue on the active site of COX-2 in the same fashion as its binding to the active site of COX-1. SER-530 is located near COX-1 active site and the Ac group added to this SER residue hinders the access of substrate (arachidonic acid, AA) to the active site [70]. COX-1 and COX-2 are isoenzymes (60% sequence identity) and their active site structure are similar too [71]. COX-2 is inducible, and its expression is enhanced by the same prostaglandins that are synthesized by COX-1 in platelets and epithelial cells [55]. However, COX-2 has a side pocket in the substrate channel that is not in COX-1. Additionally, the substrate channel of COX-2 is larger and more flexible. These structural differences make ASA (at high doses, i.e., micromolar to millimolar) about 60-fold more potent to inhibit platelet COX-1 than monocyte COX-2 (as measured on prostanoid biosynthesis by washed human platelets (expressing COX-1) and isolated monocytes (expressing COX-2)) [46]), and 170 times more effective at inhibiting COX-1 anti-inflammatory activity of a COX-isozyme activity as IC50 ratio of COX-2/COX-1 [72]. COX-2 gene expression is induced by myriad stimuli and is sustained by a positive feedback mechanism via prostaglandins (PGE2 and PGI2). These prostaglandins activate adenylyl cyclase (GC), thereby increasing intracellular cyclic AMP levels and enhancing COX-2 transcription via the protein kinase cAMP-dependent (PKA) pathway [73] (Figure 4). A third cyclooxygenase, COX-3, is selectively inhibited by low concentrations of some NSAID including ASA. Blocking or changing COX activities are the result of acetylation by the active acetate released from the ASA molecule and not by salicylate [74]. This has been shown in platelet studies, where a low concentration of ASA is sufficient to irreversibly block COX-1, while salicylate is inactive. In humans, deacetylation is so intense in the intestinal lumen, portal circulation and liver that it is assumed that only about 50% of the ASA dose reaches the systemic circulation unchanged [39,75]. Low-dose ASA provides a paradigm of COX isozyme-selective and cell-specific inhibition, by virtue of its short half-life and its ability to inactivate COX irreversibly. Other NSAIDs lack these unique pharmacokinetic and pharmacodynamic features and do not usually achieve the same degree of persistent platelet COX-1 inhibition as is obtained with low-dose ASA [76].

However, other COX independent ASA mechanisms have gradually been identified. Subsequent studies aimed to identify additional effects of this drug (such as chemopreventive or other) that could help explain its pharmacological properties. ASA can modulate many different signaling pathways. It was found to inhibit activated B cell nuclear factor κ-light chain enhancer (NF-kB) without affecting other transcription factors. It was first demonstrated in 1994 [77]. ASA inhibits the activation of NF-kB, thus prevents the degradation of the NF-kB inhibitor, I kappa B, and therefore is retained in the cytosol. ASA also inhibits NF-kB in B-dependent transcription from the Ig kappa (Igκ) enhancer and the human immunodeficiency virus (HIV) long terminal repeat (LTR) in transfected T cells [77]. It has also been demonstrated that ASA alone is capable of stimulating hippocampal plasticity. It was observed that ASA binds to PPARα at the Tyr314 residue of its ligand-binding domain (LBD).

On binding to the PPARα LBD, ASA induces activation of PPARα to upregulate transcription of the cyclic adenosine monophosphate response element-binding protein (CREB) and associated hippocampal plasticity and associated hippocampal plasticity [78]. Furthermore, low-dose of ASA treatment increased the α-amino-3-hydroxy-5-methyl-4-isoxazolepropionic acid (AMPA)-and N-methyl-D-aspartate (NMDA)-mediated calcium current in hippocampal slices and improved memory and learning in the FAD5X, but not FAD5X/Ppara-null, mice [78]. However, analgesic doses of ASA do not cause psychiatric disturbances such as hypnosis or rapid mood changes, relieve pain without affecting other sensory modalities and do not modify excitatory mechanisms involving the brainstem reticular formation. Therefore, it seems very likely that any central mechanism is probably subcortical, perhaps at the level of the hypothalamus. The small number of such effects in the CNS also suggests that the analgesic action of salicylates is largely through their peripheral action [79].

So far, many mechanisms have been proposed in which it has been proven that ASA influences transcription factors [54,80,81,82], cell signaling [83,84] and mitochondrial function [85], as well as the activity of various enzymes by many different action types (Figure 4, Table 2) [86,87]. ASA induces autophagy and stimulates mitophagy. However, not all of the exact mechanisms by which ASA works are not still known and understood.

## 4. The Potential Repurposing of ASA

Drug repurposing (also known as drug repositioning, drug reprofiling, drug redirecting, drug retasking and drug therapeutic switching) by definition is a strategy for identifying new uses for approved or investigational drugs that are outside the scope of the original medical indication [93,94]. Indeed, the drug therapeutic switching can relate to the development of existing drugs for new diseases or new patient populations, new dosage forms, routes of administration or line of treatment [95]. Comparing traditional de novo drug discovery and development with drug repositioning reveals a time reduction from 10–17 years to 3–12 years. The reposition procedure is shortened because several phases common to traditional drug discovery and development can be omitted. Candidates often went through several phases of development for their primary indication. Moreover, de novo drug discovery has a success probability of less than 10%. Drug repositioning also reduced the cost investments (from USD 12 bilion to USD 1.6 bilion) [96]. For short drug repositioning, is old drugs approved for new uses, is an effective strategy to find new indications for existing drugs and is highly efficient, low-cost with reduced risk. Due to this drug therapeutic switching has become a popular strategy in recent years. Examples of successful drug repositioning to date include sildenafil, fentanyl, thalidomide, retinoic acid, and others [97]. In past 10 years, academic researchers, pharmaceutical companies and governments have launched large-scale funding and activities to support drug repositioning-related studies.

The main problem with drug repositioning is the detection of new drug-disease relationships. Nevertheless, it is important to always consider both the risks and the therapeutic benefits of repositioning a drug [98]. Due to the multiplicity of the mechanisms of action for ASA (Figure 4, Table 2), pleiotropic applications of ASA are widely accepted [5]. ASA was originally used as an analgesic [99] and anti-inflammatory medication [100]. Since inflammation is associated with the development and progression of many diseases, many randomized controlled trials (RCTs) have shown that regular intake of ASA significantly reduces the incidence of other diseases. It should be remembered that pleiotropy refers to disorders in which many, seemingly unrelated organ systems are involved [101]. The best examples of pleotropic use of ASA are cardiovascular diseases and cancer. Due to this, ASA is the oldest example of drug repositioning; its first repositioning was in the 1980s, at low doses only, as an antiplatelet aggregation drug [102].

### 4.1. ASA for Preventing Cardiovascular and Cerebrovascular Diseases

The term CAVDs means all types of vascular diseases, including heart coronary, brain, and peripheral vascular diseases but sometimes is discuss CAVDs and CEVDs separately [103]. While CAVDs is influenced by multiple risk factors and mechanisms, acute cardiovascular events are largely triggered by thrombosis. ASA reduces the stickiness of platelets, which prevents platelets from adhering to the inside of the artery and forming a thrombus, which reduces the risk of heart attack or stroke. The anticoagulant activity of ASA was first implied by Laverence Craven in 1953 [104], and this has encouraged many researchers to conduct research in this area [105,106]. This resulted in FDA approval of the ASA in 1985 for the treatment of acute myocardial infarction and secondary prevention based on a meta-analysis of individual equivocal studies [107]. Since that date, ASA became the cornerstone of antiplatelet therapy. A set of trials was designed in the early 1990s to test the efficacy of lower doses of ASA in the prevention of CEVD ischemia [15,17]. Based on many RCTs (such as BMD [108], PHS [109], TPT [110], PPP [111], WHS [112], POPADAD [113], JPAD [114], AAA [115], JPP [116]), the FDA did not recommend ASA for the primary prevention of CAVD [117]. This was due to fact that the benefits associated with the use of ASA for prevention were doubtful at best and were associated with increased bleeding risk. It should be emphasized here that clinical trials and meta-analyses such as COMPASS [118], COMMANDER HF [119] and VOYAGER PAD [120,121] have shown that patients who experience minor ischemic strokes or transient ischemic attacks (TIAs) show benefits from antiplatelet therapy [122,123]. Additionally, studies such as HOT [124] or ASCEND [125] revealed a significant reduction in CAVD incidents. Due to these facts, the guidelines for the management of acute ischemic stroke and TIA recommend antiplatelet therapy, typically providing some recommendations for use of a single agent, most commonly ASA [122,126]. However, the recently published updated RCT PROSPERO meta-analysis: CRD42018115612 [127], based on data from RCT studies such as JPAD 2 [128], ARRIVE [129], ASCEND [125] and ASPREE [130,131], verified the efficacy and safety of ASA in the primary prevention of CAVD. Collectively, this study revealed that (1) ASA use for primary prevention decreased nonfatal ischemic events and increased nonfatal bleeding events and (2) the benefits were more pronounced when the data were estimated with a minimum follow-up of 1 year [132] (estimated risk of ASCVD was ≥7.5% in 10 years). Together, various data from RCTs and meta-analyzes have shown that the use of ASA in the primary prevention of cardiovascular disease is highly controversial [117]. It is worth noting that the FDA has never approved ASA labeling as the primary target for CAVD and as a result, ASA never changed its profile to CAVD [133]. Additionally, taking into account the latest literature reports, it seems unlikely that ASA will be reprofiled to primary treatment of CAVD. According to the latest the American College of Cardiology (ACC) and American Heart Association (AHA) Joint Committee on Clinical Practice Guidelines [134,135,136], ASA has been assigned for 2a (i.e., moderate) and 2b (i.e., weak) classes of recommendation.

### 4.2. ASA Use for Cancer Therapy

The first relation between ASA and cancer was reported in 1971 [137]. ASA is not only used for cancer prevention [138,139,140], but also administered for adjuvant cancer therapy (Figure 4, Table 2) [141,142]. Over the last several decades studies have proven that ASA can act in different types of cells such as epithelial cells, tumor cells, endothelial cells, platelets and immune cells. Therefore, ASA acts on various characteristics of cancer such as continuous tumor growth, metastasis, angiogenesis, inflammation and evasion of immune responses. There are many studies to determine the effects of ASA on tumor progression and recurrence [143]. The preclinical studies demonstrate that ASA is able to suppress tumor growth in animal models of various types of cancers [144]. The therapeutic application of ASA reduces the risk and mortality from certain cancers such as colon [145,146,147], ovarian [146,148], prostate [149], endometrial [150], hepatocellular [151], skin [152], esophageal [153], pancreatic [154], breast [155,156,157], bladder [158], head and neck [159]. Recent reports indicate that daily ASA use, whether regular strength or low dose, results in reductions in cancer incidence and mortality [160,161] and also prevents distant metastasis [162,163].

The major outcome measures in many clinical trials include biomarkers such as NF-kB, tumor size, cancer recurrence or metastasis and disease-free survival or overall survival [164]. Colorectal cancer (CRC) is one of the most common malignancies in the world. In addition, some of clinical trials focus on specific subtypes of colorectal carcinoma, such as PIK3CA-mutated colon cancer. Reductions in prostacyclin (PGI2) due to COX-2 inhibition in the vasculature, thus reducing vasodilation with a consequence increase in thromboxane, were proposed as the underlying mechanism for this increased risk [77,145]. The secretion of PGE2 by tumor cells suppresses NF-κB, which plays a role in inducing the natural killer (NK) cells, thus preventing these immune cells from maturing and reducing their ability to damage cells [165,166]. Prolonged treatment of CRC cells with ASA decreases cytoplasmic IκBa and thus increases translocation of NF-κB to the nucleus; such activation of the NF-κB pathway induced apoptosis in these cells [167] (Figure 4).

### 4.3. ASA as Promising Candidate for Repositioning in the Treatment of Severe Bacterial Infections of MDR Bacteria, Extensively Resistant (XDR) and Pan-Resistant (PDR) to Drugs

Anti-inflammatories are drugs used to reduce inflammation, which involve two primary objectives, pain relief and delaying or arresting the process responsible for tissue damage. Antimicrobial activity of ASA resulted in MIC values above 1000 µg/mL in strains of Gram-negative and Gram-positive bacteria [168]. When ASA was associated with pyrazinamide, in a study carried out in vivo, a reduction in the bacterial count in the lungs and in the spleen of mice was demonstrated, suggesting that this drug could induce a low metabolic state in the cell, which would increase its susceptibility to antibacterial agents [169]. Although, other work reported that their performance would be through the alteration of the permeability of the outer membrane proteins (OMP) or by the rupture of the bacterial membrane [170].

### 4.4. ASA Potential for Neuropsychiatric Disorders

In recent decades, it has been proven (in neuropathological, experimental and animal studies) that NSAIDs reduce the risk of developing neuropsychiatric disorders [171,172]. Of note, however, the exact organic nature of these deficits is difficult to pinpoint [5]. A chronic inflammatory response is characterized by activated microglia, reactive astrocytes, complement factors and increased inflammatory cytokines [173,174]. Indeed, microglia are specialized macrophages localized to the CNS that also play an important regulatory role in the inflammatory response [175]. It should be emphasized here that microglia derive from myeloid precursors that enter the developing CNS to become the major population of brain resident macrophages [176]. In the CNS, COX-1 is constitutively expressed in neurons, astrocytes and microglial cells. COX-2 is upregulated in these cells under pathophysiological conditions [174]. COX-2 is upregulated and plays pathophysiological roles in long-term potentiation (LTP), stroke, Alzheimer’s disease (AD), Parkinson’s disease (PD), multiple sclerosis (MS), amyotrophic lateral sclerosis (ALS), epilepsy, schizophrenia (SCH), bipolar disorder (BD), Creutzfeldt–Jakob disease (CJD), acquired immunodeficiency syndrome (AIDS) and neoplasm [172]. One study provides an important and timely review of preclinical and clinical studies investigating the use of COX-2 inhibitors across multiple psychiatric disorders including major depressive disorder (MDD), SCH, BD, autism spectrum disorder (ASD) and obsessive–compulsive disorder (OCD) [175].

On the other hand, ASA, a widely used analgesic, binds to the LBD domain of PPAR. and upregulates hippocampal plasticity via PPAR. After oral administration, ASA improves hippocampal function and protects spatial learning and memory in an animal model of AD via PPAR. Therefore, low-dose aspirin may find therapeutic use in AD as well as in other dementia-related illnesses.

It is also important to mention that in the central nervous system (CNS), physiological levels of pro-inflammatory cytokines, such as interleukin-1 and -6 (IL-1, IL-6) and tumor necrosis factor α (TNF-α), are involved in neural processes such as the induction and maintenance of long-term potentiation, neurogenesis, regulation of the survival of differentiated neurons and the development of astrocytes, impacting on several cognitive (e.g., memory) functions [177]. Nonetheless, in response to several injuries, such as ischemia, trauma and neurotoxicity, cytokines may contribute to the neurodegenerative process. ASA, due to its anti-thrombotic properties, is useful in the prevention of deterioration in cognitive function caused by ischemia.

### 4.5. ASA Use for COVID-19

At the present time, the drug repositioning approach has taken on a new urgency due to the worldwide Coronavirus disease (COVID-19) epidemic [178,179]. Repurposing ASA to treat hospitalized COVID-19 patients seems to be a sensible approach [180,181]. ASA is well known for its anti-inflammatory, analgesic, and anticoagulant properties. ASA has been suggested to be an antiviral agent due to its activity against DNA and RNA viruses (Figure 4) [170], especially since ASA has been reported to be able to reduce RNA synthesis and replication of human coronavirus-299E (CoV-229E) and Middle East Respiratory Syndrome (MERS)-CoV in one in vitro study [182]. However, the use of ASA in COVID-19 is still controversial due to the opposite study results. For example, Chow et al. showed that the use of ASA was associated with improved treatment outcomes among hospitalized COVID-19 patients [183], while Yuan et al. and Sahai et al. reported otherwise [184]. Given that many clinicians are already using ASA to treat viral infections, including COVID-19 off-label, without referring to solid evidence of safety or efficacy, there is an urgent need for well-conducted, randomized clinical trials in this area. The results of such studies will help guide clinical practice [185].

## 5. ASA Dose and Formulation

ASA inhibition of inflammatory cell function requires a higher and more frequent dosage whereas very low doses of ASA inhibit platelet aggregation and platelet thromboxane production.

The pharmacological actions of ASA are determined by three compounds: ASA, Ac and SA. SA is formed from its precursor ASA within 15–20 min after oral application and is responsible for the anti-inflammatory, antipyretic and analgetic activities of ASA.

At high concentrations, ASA has been shown to react with nucleophilic groups on proteins resulting in irreversible acetylation (Table 2, [53]). For example, when dose-dependent inhibition of prostaglandin biosynthesis was excluded as the only mechanism of action for ASA, other mechanisms could be elucidated. In the case of the antiplatelet effect of ASA (with low dose) as a disruption of COX 1–2 function, it was found that ASA (in high dose) acetylates the residual lysine in fibrinogen, resulting in increased permeability of the fibrin clot and increased lysis of the clot [5,179]. In this way, it was proven that ASA reduces the production of thrombin by directly promoting fibrinolysis and, consequently, attenuating the clotting reactions mediated by thrombin [180]. Another mechanism of the antiplatelet action for ASA has also been proven, in which the activation of platelets with neutrophils is inhibited by the use of nitric oxide and cGMP [181]. Acetylation of other proteins may account for a variety of additional effects, e.g., anti-cancer activity. Table 3 summarizes the dose-dependent effect of ASA in various mechanisms.

The therapeutic effect of ASA is determined not only by the dose, but also by the form of its administration [5]. It is worth noting that the use of multiple drug delivery systems creates additional therapeutic benefits for the patient. Such benefits are mainly related to the increase in solubility, degree of ionization and surface area. Formula modifications significantly affect the pharmacokinetics of ASA through the ability to transport the drug directly to the desired site of action in the body and by reducing the side effects of the drug. The use of various forms of ASA has been confirmed by studies, shortening the time to the clinical onset of analgesia. Currently, ASA is used based on every possible route of administration, i.e., oral, rectal (uniform suppositories US-ASA), injection (Iv -ASA), transdermal (T-ASA) and inhalation (I-ASA) [198]. The oral ASA delivery system includes various forms, such as regular (or plain) tablets (RT), gastro-resistant tablet (GR, also called enteric-coated (EC)), effervescent tablets (ET), modified release forms (MR), chewable tablets/gum (ChG-ASA), dispersible ASA tablets (D-ASA) and granules (G-ASA).

With long-term administration of ASA, the major side effect is ASA-induced gastric trauma, which increases the risk of gastric ulcers and gastric bleeding. Mitigating the side effects of ASA on stomach tissue has become an extremely important and competitive area of research. The mechanisms of ASA-induced gastric damage are complex. Generally, we can classify them into two categories. The first involves a prostaglandin-dependent mechanism that inhibits prostaglandin production through the ASA inhibition of COX. The second mechanism is prostaglandin independent, involving apoptosis of the gastric epithelial cells due to the accumulation of ASA in the cells. The mucosal barrier is compromised, infiltrated immune cells would release a series of pro-inflammatory cytokines to induce an immune response, causing inflammation and exacerbating damage to the gastric mucosa. For this reason, strategies have been developed to alter the route of administration of ASA (e.g., transdermal, inhalation and rectal administration).

*Transdermal ASA (T-ASA):* Transdermal drug delivery (TDD) is a systemic delivery method that can deliver drug through the skin into the systemic circulation at a constant rate over a long period of time. TDD systems are believed to be the most attractive of topical therapies due to their low failure rate, ease of distribution, excellent efficacy and user preference [199]. TDD has gained attention recently as it can bypass the gastrointestinal tract and is painless, especially with prolonged use, unlike classical drug delivery methods such as oral administration and a hypodermic needle. It is noteworthy that only drugs suitable for transdermal administration fall within a narrow range [200]. They are characterized by a low molecular weight (<500 Da) and high oil–water partition coefficients (log P > 1.5) [4]. To date, around 20 active pharmaceutical ingredients have been developed and approved by the FDA as transdermal products, all of which are small molecule lipophilic drugs. The physicochemical properties of ASA that make ASA a good candidate for the formulation of transdermal patches are MW = 180.16 Da, log P = 1.2 and an aqueous solubility of 3 mg/mL [199]. The normal physiological pH of human skin is between 4.7 and 5.6. which allows deprotonation of the carboxylate group and increases the rate of both hydrolysis and transacetylation. Low bioavailability of ASA (a plasma half-life of 15–20 min) through the skin and avoiding direct contact with COX-1 expressed on the cells of the gastric mucosa can be ensured, inter alia, by the safer way to stop the blood platelets from working [201].

T-ASA is useful not only in medicine but also in the skin care sector. It is mainly used in cosmetics and helps in healing pimples and irritations. It dissolves in fats; therefore, it exfoliates dead cells from the surface of the epidermis and penetrates the sebum layer. It penetrates deep into the skin and hair follicles. It cleans the pores of the skin and has an anti-comedogenic effect, which is important in the treatment of acne and pimples. It tightens the skin and has a whitening effect. Cosmetics with ASA include a mask and a tonic. The ASA mask has a disinfecting and anti-inflammatory effect, which is why it also soothes inflammation of the skin and dries purulent eczema. It softens and evens out the color. It also smooths the complexion. The ASA tonic exfoliates the skin and preserves the effects of the mask. It is used to fight blackheads. Cosmetic preparations based on ASA are recommended for skin prone to contaminated pores and acne. It can also be used by people with eczema, but also by those with blackheads or people with combination skin. A contraindication is sensitive and couperose skin. The ASA mask should also not be used by people with open wounds or by those with an advanced form of acne, pregnant women and people allergic to ASA.

*ASA inhalable formulation (I-ASA):* The I-ASA formulation has several important advantages, such as rapid absorption of the drug due to the high permeability and large absorption area in the lungs (approximately 70–140 m^2^ in human adults who have very thin mucosa, pH ∊ (7.35–7.45). In addition, good blood supply to the lungs ensures rapid exchange. Moreover, first-pass metabolism is avoided [202]. A non-invasive way of administering therapeutic agents is achieved by using dry powder inhalers (DPIs). DPIs are devices by which an active drug in dry powder form is delivered through the pulmonary route to produce a local or systemic effect [203]. I-ASA is a recent patent that described the development of inhalable ASA in dry powder inhaler for rapid absorption [204]. In a phase 1 study, compared to ingested and swallowed ASA, I-ASA provided earlier and greater exposure to the drug and was a faster way to achieve greater platelet inhibition [205]. This form of ASA administration is particularly important in the treatment of cardiovascular disorders.

*Uniform Suppositories of ASA (US-ASA):* The physiological pH of the liquid filling the rectum fluctuates within the pH ϵ range 7.6–8.0. However, pathological states change the pH and thus inflammatory changes of the mucosa increase its acidity (to pH = 6.6–6.5), whereas neoplastic changes cause alkalization of the liquid filling the rectum to pH ∊ 7.8–8.4. Through the mucosa of the rectum, it penetrates and dissolves non-dissociated and lipophilic substances. Therapeutic indication of the US-ASA formulation consists of mild to moderate pain (including headache, neuralgia and sore throat); pyrexia (as in colds and influenza); pain and inflammation in rheumatic disease, arthritis and sciatica [90,91].

The absorption of ASA when administered orally is rapid and complete. The absorption is directly proportional to the dose and, thus, follows first order kinetics. Absorption takes place, to a minor extent, in the stomach and, to a larger extent, in the upper small intestine [52].

*Regular tablets (R-ASA, pills, plain ASA)* Plain, uncoated, R-ASA can be used for the (*) treatment of mild to moderate pain including headache, migraine, backache (including lumbago), nerve pain (neuralgia and sciatica), period pain, toothache and sore throat. (**) It can be used for symptomatic relief of colds and influenza. (***) It is used for the relief of rheumatic pain (arthritis), muscle tenderness and stiffness (including fibrositis), muscular aches and pains, the pain of joint swelling and stiffness, strains and sprains. (****) It is effective at the reduction of high temperatures. [70,89]. R-ASA is rapidly absorbed from the acid environment of the stomach and upper intestine. The oral bioavailability of R-ASA tablets is approximately 40–50% over a wide range of doses. However, orally administered ASA requires high and frequent dosing because it undergoes extensive pre-systemic hydrolysis in the gut and liver into SA which is devoid of antiplatelet activity. Continuous exposure of new platelets to ASA is necessary to achieve prolonged inhibition of platelet aggregation.

*MR forms* are mainly divided into two classes: extended-release (ER) and delayed-release (DR) forms of ASA. Commonly, drugs in MR forms are administered in tablets and capsules. The goal of designing MR forms is to reduce the frequency of dosing or increase the effectiveness of the drug. This is accomplished by localizing the drug to the desired site of action, reducing the required dose, or providing uniform drug delivery (i.e., achieving a steady state of drug in the blood over an extended period of time). The reduced dosing frequency minimizes side effects and increases patient compliance, making pharmacotherapy safer. However, the most serious drawback of this drug formulation is the inability to immediately discontinue therapy and the significantly less flexible adjustment/dose modification [206]. MR dosage forms allow the rate and extent of drug absorption to be controlled to accommodate therapy goals in the treatment of chronic diseases. ER drug products are dosage forms that release the drug slowly over a longer period of time compared to regular dosage forms. They therefore allow for prolonged absorption of the drug and increase the rate and extend of the drug’s presence in the blood (bioavailability) so that its plasma concentration remains within the therapeutic range for a longer period of time. In contrast, DR drug products are dosage forms that release some of the drug at times other than immediately after administration. DR-ASA is used to protect the stomach from its irritating effects. The ASA tablet is coated with an acidic material to reduce the release of the drug in the stomach and to allow the drug to be released and dissolved in the small intestine. The principle of the DR-ASA drug is based on the control of pH function. ASA in the stomach (pH ∊ (1–3)) is in a nonionized state, which means greater lipophilicity and better passage through the semipermeable lipid bilayer membrane of the stomach. However, it has been shown that ASA is actually absorbed mainly from the intestinal wall (pH ∊ (6.5–8) and not from the stomach. This is because for a drug to be absorbed from the gastrointestinal tract, it should have adequate hydrophilicity and optimal lipophilicity (logP = 1.2 at pH = 2 in the stomach; logP = −1.9 at pH = 7 in the intestine).

*GR-ASA* or *EC-ASA* forms are commonly classified as one type of DR products. The reason behind the development of EC-ASA was an effort to reduce the number of ASA side effects on the gastric mucosa [207]. Under physiological conditions, ASA is very quickly hydrolyzed to SA, 70–90% of which is bound to plasma proteins. Increased pH of intestinal fluid results in increased ASA dissociation, thus slowing down the rate of its absorption [51]. GR-ASA formulations now dominate chronic use, especially in North America, for secondary prophylaxis of thrombotic CAVD, CEVD and after bypass surgery [208]. They are used, among other things, in the secondary prevention of myocardial infarction, prevention of cardiovascular disease in patients with stable angina, documented history of unstable angina (except acute phase), prevention of vascular graft obstruction after coronary artery bypass grafting (CABG), coronary angioplasty (except acute phase), secondary prevention of transient ischaemic attack (TIA) and ischaemic incidents, use of a multidisciplinary approach to the treatment of cerebral ischaemic attack and ischaemic cerebrovascular accidents, provided intracerebral haemorrhage is excluded [209]. Polymer materials for drug coating these applications are generally characterized by good water solubility. In contrast, water-insoluble polymers are typically used to achieve sustained drug release over an extended period of time [210]. Common water-soluble materials include hydroxypropyl methylcellulose (HPMC), polyvinyl alcohol, hydroxypropyl cellulose, polyvinylpyrrolidone-vinyl acetate copolymer and polyvinyl alcohol-polyethylene glycol copolymer. Examples of polymers used in achieving sustained drug release include ethyl cellulose, polymethacrylate copolymers with quaternary ammonia groups and polyvinyl acetate. GR-ASA results in its release into the alkaline environment of the small bowel, where it is hydrolyzed. However, GR-ASA has lower bioavailability than regular ASA. Nonetheless, the antiplatelet effects of full-dose (>300 mg) enteric-coated ASA are similar to those of uncoated formulations. On the other hand, the efficacy of low-dose (<100 mg) GR preparations has not been clearly established, and it is possible that such doses may result in inadequate platelet inhibition. Thus, if coated ASA is prescribed, larger doses may be necessary to obtain the desired antiplatelet effect [211].

Currently available data suggest that GR-ASA does not exert a demonstrably protective effect on the gastric mucosa. Additionally, in light of the newest data, the GR-ASA effect in the secondary prevention of arterial thrombosis is unclear. The implication is that no GR formulation, but only plain ASA absorbing in the stomach should be used in the treatment of coronary heart disease as a sufficient body of evidence supporting its efficacy has been gathered for this formulation only [210,212].

*Chewing gum (ChG-ASA):* Medicated chewing gum consists of a chewing gum core coated with a coating that may be a layer of polymers, waxes, sweeteners, sugar, flavors or colors. Chewable tablets are compressed dosage forms that are chewed in the mouth before swallowing. The pharmacologically active ingredient may be present in the core, the rim or both. Medicinal chewing gum is a very convenient way of dosing the drug. This form of the drug is primarily intended for specific groups of patients who have difficulty swallowing, such as children or the elderly. During chewing, the medication from the gum is released into saliva (saliva pH ranges from pH ∊ 5.8–7.1). The released drug has two routes of absorption: through the oral mucosa or it can reach the stomach for absorption through the gastrointestinal tract. The drug absorbed directly through the buccal mucosa prevents metabolism in the gastrointestinal tract. The trade name of the American analgesic chewing gum containing ASA as the active ingredient is *Aspergum*, and it was introduced in 1928 [213]. This chewing gum is still available and contains 227 mg of ASA in cherry and orange flavors [214]. In 1991, the European Pharmacopoeia defined the intended use of medicated chewing gum as a topical treatment for oral diseases or for systemic delivery after absorption through the buccal mucosa or from the gastrointestinal tract. Ch-ASA is used for its analgesic, anti-inflammatory and antipyretic properties. It is also worth noting that the use of low-dose ASA as an anticoagulant in the prevention of heart attacks and strokes is historically associated with the form of ChG-ASA. Research on anticoagulant properties was initiated by the observation of Lawrence (in 1953), in which he found that many of his tonsilectomy patients who had used ChG-ASA to relieve pain had to be hospitalized for bleeding [104]. Paradoxically, it is these incidents related to the use of ChG-ASA as an analgesic that contributed to the use of low doses of ASA in the prevention of CAVD [215].

*Effervescent tablets (ET-ASA):* ET-ASA tablets are prepared by thickening and contain, in addition to the active pharmaceutical ingredient(s), mixtures of acids (e.g., citric or tartaric acid) and carbonates and/or bicarbonates. Upon contact with water, these formulations release carbon dioxide, producing a characteristic effervescent effect [52]. A popular product in this group is Aspirin C^®^ containing ascorbic acid (vitamin C, AA). The addition of vitamin C is beneficial in the course of colds, during which there is an increased need for it by the body. Moreover, the co-formulation of ASA with AA is considered to mitigate and ameliorate reactive oxygen species (ROS). Monotherapy with ASA has been shown to decrease the concentration of vitamin C not only within the intestines but also in gastric juice. It seems that the ROS-induced gastrointestinal damage could also be also caused by the negative impact of ASA therapy through reducing the ability of intestinal mucosal cells to manage oxidative stress. Additionally, the extent of ionization of ASA and AA as weak organic acids differs in the intestinal lumen, consequentially affecting their ability to access transporters in the gut wall. Indeed, it is also conceivable that the co-administration of ASA and AA may potentiate ASA serum levels and its analgesic effects [216]. Indications for using this medicine are the symptomatic treatment of pain of low and/or moderate intensity (e.g.: headache, toothache, muscular pain). Symptomatic treatment of pain and fever in the course of cold and flu [217].

*Dispersible ASA tablets (D-ASA):* D-ASA tablets should be stirred into a small amount of water. It is important that this type of aspirin tablet is taken with something to eat. This helps to reduce the risk of any irritation to the stomach. D-ASA in 75 mg dosages are indicated for the secondary prevention of thrombotic CAVD, CEVD and following by-pass surgery [209]. D-ASA in 300 mg can be used to treat headaches, migraine, neuralgia, toothache, sore throat, period pains and for the relief of sprains, strains, rheumatic pain, sciatica, lumbago, muscle aches, fibrositis, joint swelling and stiffness [218].

*Fast-release tablets:* Rapid achievement of therapeutic plasma levels of the drug is one of the basic conditions for the rapid action of analgesic drugs. The early onset of drug action has also been shown to be important in the symptomatic treatment of acute pain such as a migraine attack [219]. Orally disintegrating tablets (ODTs) disperse or melt within seconds on coming in contact with saliva and thus overcome swallowing problems [220]. This formulation is typically characterized by a reduction in particle size (in the form of granules, G-ASA) thereby increasing the surface area available for absorption, and the addition of sodium carbonate which acts as both a disintegrant and a local buffer [221]. By definition, the granules are solid dosage forms that are composed of agglomerations of smaller particles. Oral dissolving granules (orodispersible) are very simple to apply because they must be placed on the tongue and allowed to dissolve. In general, the immediate-release ASA is used in acute settings. Additionally, compared to conventional ASA tablets, an improvement of time to meaningful pain relief by the factor of 2 has been demonstrated (49 min vs. 99 min) [50].

*Injection form of ASA (IV-ASA):* Due to the fact that oral administration of non-narcotic analgesics is often not possible immediately after surgery, it is common practice to administer these drugs by injection. As early as 1974, Kweekel de Vriesat et al. proved that postoperative pain control (in the immediate postoperative period) by 1 g of intravenous ASA is as good an analgesic as 10 mg of morphine [222]. Typically, a parenteral salicylate preparation, the injection of ASA is administered in the form of lysine acetylsalicylate (LAS). LAS is a water-soluble salt of ASA and therefore allows intramuscular and intravenous injections [223]. Shortly after being administered, LAS is converted into ASA, which is metabolized in the liver to SA (active form) [224]. Accordingly, this has a pharmacological effect similar to that of ASA [225]. In the LAS formulation, 1.8 g of powder prepared by lyophilization is equivalent to 1 g of T-ASA [226]. LAS, at the recommended oral dosages ranging from 160 to 325 mg/day, is a powerful antiplatelet compound with fewer gastrointestinal side effects than R-ASA and EC-ASA, and has proven effective in reducing platelet activation [227,228]. In subsequent years, intravenous LAS has been shown to be effective in the treatment of acute migraine attacks. Additionally, LAS has been shown to be effective and well tolerated in the treatment of severe rebound headache in patients undergoing drug withdrawal [229].

In sum, the availability of a variety of ASA formulations as painkillers provides clinicians and patients with a variety of options depending on drug therapy preferences and pain characteristics.

## 6. ASA Derivatives

The use of ASA derivatives dates back thousands of years. Therefore, many different ASA derivatives are known and studied. Selected groups of ASA derivatives are briefly discussed below.

**Figure 5:**
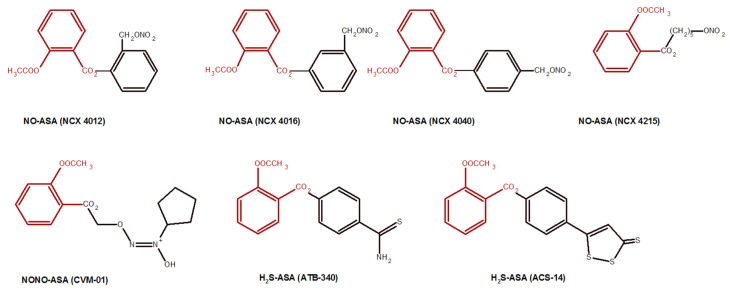
Chemical structures of NO-ASA and H_2_S-ASA derivatives. ASA moiety is marked in red.

*Nitric oxide-donating (NO*) *compounds* represent a novel emerging class of pharmaceutical agents. The pharmaceutical company has produced compound [230], which combine ASA with a NO-releasing moiety. NO itself is a potent vasodilator and inhibitor of leukocyte adhesion to the gastric vascular endothelium [231]. Due to this, the NO liberated in the stomach protects the stomach mucosa from damage by gastric hydrochloric acid [66]. In nitric oxide-donating aspirin (NO-ASA), consisting of traditional ASA which contains a NO-releasing moiety (-ONO_2_), the -ONO_2_ is covalently linked with ASA by a spacer. Additionally, H_2_S-aspirin (H_2_S–ASA) hybrids, compounds with double bond sulfur moiety, have a wider spectrum of action and fewer gastrointestinal side effects. Furthermore, hypertensive effects of using NSAIDs could be reduced by the oral administration of H_2_S–ASA derivatives [232,233] (Figure 5).

In light of recent studies, NO-ASA appears to be safer than ASA and is currently undergoing clinical evaluation for the prevention of colon cancer [233]. Compared to NO-ASA to ASA, NO-ASA was found to be >1000 times more potent in inhibiting the growth of the colon and other cancer cell lines [234,235]. NO-ASA inhibits proliferation and induces apoptosis in colon cancer cells, and these effects may explain its remarkable potency enhancement [235].

Although aspirin has promising effects on preventing colorectal cancer, the combination of aspirin and other natural agents may be more effective in preventing carcinogenesis [143].

**Figure 6:**
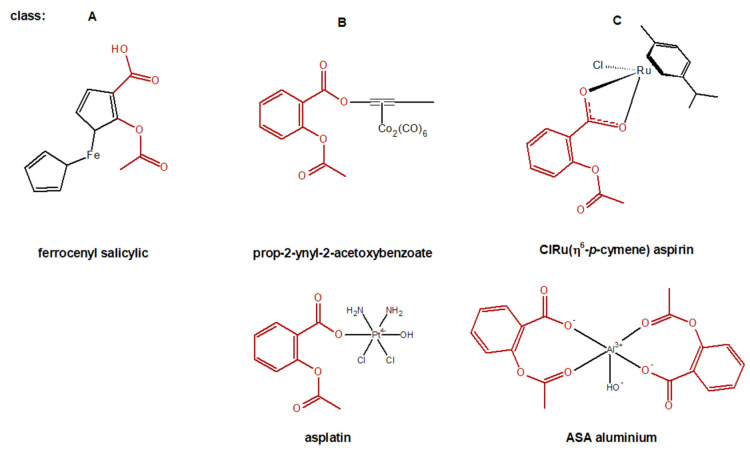
Selected chemical structures of general organometallic derivatives of ASA. Classification according to [236]. ASA moiety is marked in red.

*Organometallic ASA derivatives:* General organometallic derivatives of ASA can be structurally divided into three classes: A, B and C. Class A contains compounds in which the benzene ring of ASA is completely substituted by an organometallic group. Derivatives in which the metal group is peripherally connected to the ASA core by a suitable linker are classified as class B. Class C compounds, on the other hand, are systems in which the ASA core is coordinated to the organometallic ligand by one or two oxygen atoms (Figure 6) [236].

ASA complexes with various metal ions (Fe, Co, Rh, Mn and Mo) have shown promising anticancer activity, e.g., against MCF7 to the tune of the IC50 value in the μM range [237]. ASA with the hexacarbonyl dicobalt complex Co_2_(CO)_6_ forms the leading compound ([prop–ynyl–2–acetoxybenzoate]hexacarbonyldicobalt, Co-ASS) for a class of antiproliferative agents [238]. The Asplatin, a Pt (IV) prodrug of cisplatin containing the ASA moiety, which exhibits significantly higher cytotoxicity than cisplatin towards tumor cells and almost fully overcomes drug resistance in cisplatin-resistant cells [239].

**Figure 7:**
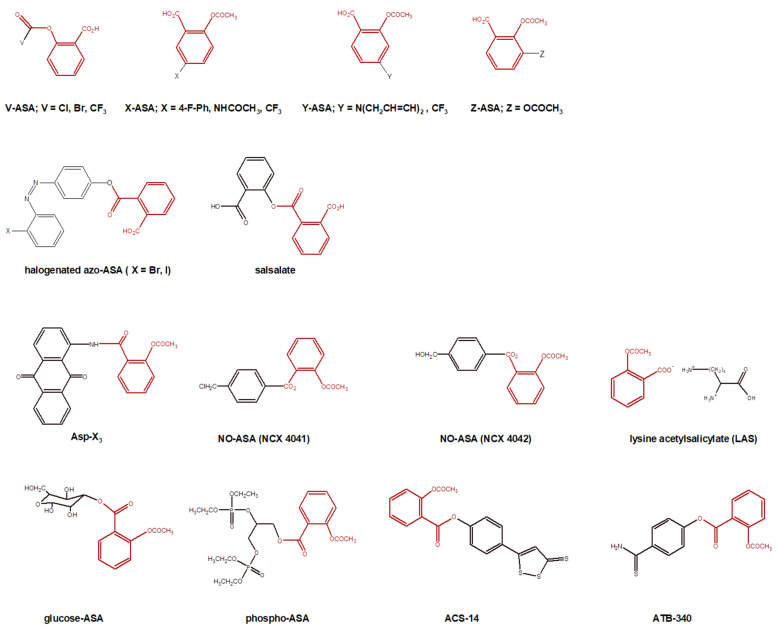
Chemical structures of other ASA derivatives. ASA moiety is marked in red.

*Other ASA derivatives* (Figure 7). Addition to Ph ring of new functional groups (–CH_3_, –OCH_3_, –NH_2_, –CH_2_NH_2_, –NHCOCH_3_, –OH, –CH_2_OH, –F, –CF_3_) not only improved the physicochemical properties but also increased the binding affinity and specialty. The calculated binding affinity of ASA is pKi = −6.5 compared to Z-ASA (where X = CF_3_) pKi = −7.2; Y-ASA (where Y = NHCOCH_3_ or CF_3_) pKi = −7.0; pKi = −7.2; Z-ASA (where Z = NH_2_) pK_i_ = −5.8 [240]. Compounds azo-ASA exhibit better antibacterial activities against both Gram-negative and Gram-positive bacteria than the reference ampicillin and aspirin alone. The presence of reactive groups of –OH, N=N, C=O and halogens significantly contributes excellent interaction of *E. coli* (MIC 75–94 ppm) and S. aureus (MIC 64–89 ppm) compared to ampicillin (MIC 93 and 124 ppm, respectively) [241]. Salsalete has anti-inflammatory, analgesic and antipyretic actions similar to ASA. It has been available for several decades, but is not commonly used. Current indications include treatment toward reduction of pain, swelling and joint stiffness caused by osteoarthritis, or other rheumatic disorders [65]. COOH group modifications contribute to lower acidity of ASA resulting in lower gastrointestinal toxicity of ASA. If OH moiety of ASA has been replaced by the diethyl phosphate moieties, respectively, the obtained derivatives are just as potent as NO-ASA, if not more, in inhibiting the growth of colon cancer cells. Glucose-ASA is seven-fold more water soluble and about eight- to nine-fold more active in inhibiting cancer cell growth than ASA [143]. The anticancer effect of anthraquinone (Asp-X3) [242] and NCX 4041 and NCX 4042 [234,243] compounds has been confirmed. Lysine acetylsalicylate (LAS) is water-soluble, making it useful for parenteral (i.m. and i.v.) administrations [244]. The administration of intravenous LAS resulted in a significant reduction of platelet reactivity compared with oral aspirin on prasugrel-inhibited platelets [224].

## 7. Conclusions

The ASA has become a milestone in two important fields: pharmacy and medicine. For a pharmacist, ASA is a long-used drug for which individual indications are practically maintained. For a doctor, ASA is primarily an antiplatelet drug that saves millions of lives of patients with coronary heart disease. These facts do not exempt us from improving therapeutic methods based on ASA, the main goal of which is to reduce the risk of side effects, as well as to extend effectiveness. Modified acetylsalicylic acid molecules already seem to be a promising therapeutic option.

## Figures and Tables

**Table 1:** The basic physical, chemical and biological properties of ASA compared to selected NSAIDs.

Solvent	Solubility of ASA at 298 K, g/100 g [a]	Solubility of SA at 298 K, g/100 g		pKa	IC_50_ *	
					COX-1	COX-2
H_2_O	0.46 [d,e]	0.22 [f]	ASA	3.5	1.67 [b]	278 [b]
EtOH	20	32.54 [f]	SA	3	>100.00 [c]	14.08 [c]
n-C_8_H_17_OH	3	1.25 [g]	paracetamol	9.7	42.23 [c]	10.69[c]
MeOH	33	38.46 [h]	ibuprofen	4.4	5.9 [c]	9.9 [c]
CO(CH_3_)_2_	29	33.33 [h]	diclofenac	3.9	0.26 [c]	0.01 [c]
AcOH	12	7.59 [h]	ketoprofen	3.9	0.11 [c]	0.88 [c]
CHCl_3_	6	2.22 [h]	piroxicam	6.3	2.68 [c]	2.11 [c]
O(Et)_2_	5	32.82 * [h]	celecoxib	11.1	15 [b]	0.04 [b]

[a] [43], [b] [44], [c] [45], [d] [46], [e] [47], [f] [48], [g] [49], [h] [42]. * Concentration of Drug (IC_50_) that inhibited 50% of Cyclooxygenase (COX).

**Table 2:** Compilation of different therapeutic targets and their action type for ASA [2,3,70,88,89,90,91,92].

Drug Targets Name	Action Type
Prostaglandin G/H synthase 1, Prostaglandin G/H synthase 2, Aldo-keto reductase family 1 member C1, Endothelin-1 receptor, Ribosomal protein S6 kinase alpha-3, NF-kappa-B inhibitor alpha, tumor necrosis factor-inducible gene 6 protein, Caspase-1, Caspase-3, Solute carrier family 22 member 6, Solute carrier family 22 member 8	Inhibitor
5′-AMP-activated protein kinase,	Activator
Cellular tumor antigen p53, Cytochrome P450 2C19, P-glycoprotein 1,	Inducer
Cytochrome P450 2C9, UDP-glucuronosyltransferase 1–6, Arylamine N-acetyltransferase 2, P-glycoprotein 1	Substrate
78 kDa glucose-regulated protein,	Binding
P-glycoprotein 1	Modulator
Tumor necrosis factor-inducible gene 6 protein, Caspase-1, Caspase-3, G1/S-specific cyclin-D1, Myc proto-oncogene protein, Proliferating cell nuclear antigen, Cyclin A,	Downregulator

**Table 3:** ASA dosage dependent action on various therapeutic goals.

Dose/Concentration Ranging of ASA	Therapeutic Goal		Cellular Concentration of ASA Need for Therapeutic Effect
	COX Dependent	COX Independent	
70–150 mg/day [55,186,187,188,189]	(COX-1) platelet effect antiplatelet	NF-κB	0.05–5 mM [54,77]
325–600 mg/4–6 h [55,189]	(COX-1, COX-2) megakaryocyte analgesic	Acetylation of p53, Glucose-6-Phosphate Dehydrogenase and Other Proteins	≥100 μM [190,191]
1.2 g/4–6 h [55,189]	COX-2) endothelial/stromal anti-inflammatory	c-Myc, Cyclin A2, CDK2 and Wnt/–catenin pathway	0.5–2.5 mM [192,193]
Single dose of 500 mg/day [194]	Fibrinolysis antiplatelet	mTOR	5 mM [195]
≤150 mg/day [196]	Activation of the gene encoding SSAT (spermidine/spermine N1-acetyltransferase) antitumor	ODC	20–100 μM [197]

## Data Availability

Not applicable.
